# A new isolation method for bacterial extracellular vesicles providing greater purity and improved proteomic detection of vesicle proteins

**DOI:** 10.1002/jex2.84

**Published:** 2023-04-25

**Authors:** Lena Hoang My Le, Joel R. Steele, Le Ying, Ralf B. Schittenhelm, Richard L. Ferrero

**Affiliations:** ^1^ Centre for Innate Immunity and Infectious Diseases Hudson Institute of Medical Research Clayton Victoria Australia; ^2^ Biomedicine Discovery Institute, Department of Microbiology Monash University Clayton Victoria Australia; ^3^ Monash Proteomics and Metabolomics Facility Department of Biochemistry and Molecular Biology Monash University Clayton Victoria Australia; ^4^ Department of Molecular and Translational Sciences Monash University Clayton Victoria Australia

**Keywords:** bacterial extracellular vesicles, *Helicobacter pylori*, outer membrane vesicles, proteomics

## Abstract

Contaminants within cell culture media often co‐isolate with eukaryotic extracellular vesicles (EVs) thus affecting their biological properties. It has yet to be investigated if this is also true for bacterial EVs (BEVs), especially for organisms grown in complex culture media containing animal‐derived products. To address this question, we isolated BEVs from the fastidious bacterium *Helicobacter pylori* grown in either standard Brain Heart Infusion (BHI) medium or BHI depleted of animal‐derived products (D‐BHI). We show that BEVs prepared from bacteria grown in D‐BHI medium have similar morphologies, size ranges and yields to those prepared from standard medium. Similarly, no differences were found in the ability of *H. pylori* BEVs to induce IL‐8 responses in epithelial cells. However, *H. pylori* BEVs prepared from D‐BHI medium were of higher purity than those prepared from standard medium. Importantly, proteomic analyses detected 3.4‐fold more *H. pylori* proteins and 10‐fold fewer bovine‐derived proteins in BEV samples prepared from D‐BHI rather than the standard method. Fifty‐seven *H. pylori* proteins were uniquely detected in BEV samples prepared from D‐BHI. In conclusion, we have described an improved method for BEV isolation. Furthermore, we demonstrate how animal‐derived products in bacteriological culture media may adversely affect proteomic analyses of BEVs.

## 1 INTRODUCTION

Cells belonging to all three domains of life release spherical membrane‐bound nanostructures, known as extracellular vesicles (EVs) (Gill et al., 2019). The EVs produced by bacteria (BEVs) are 20–400 nm in size and contain a range of products, such as proteins, nucleic acids, lipopolysaccharide and peptidoglycan, which may contribute to infection and to horizontal gene transfer (Gao & van der Veen, 2020; Kaparakis‐Liaskos & Ferrero, 2015)

BEVs are generally isolated by either ultracentrifugation or ultrafiltration, though both techniques result in relatively crude preparations (Klimentová & Stulík, 2015). Ultracentrifugation has been reported to promote vesicle and extra‐vesicle protein aggregation (Linares et al., 2015), whereas ultrafiltration results in the concentration of large protein multimers from the parent bacteria (Reimer et al., 2021), composed of flagella, GroEL and haemolysin (Hong et al., 2019). The presence of such contaminants in BEV preparations for vaccine or therapeutic applications may induce unwanted detrimental effects in the subject (Klimentová & Stulík, 2015).

Density gradient centrifugation (DGC) and size exclusion chromatography (SEC) have increasingly been introduced into EV purification workflows, with the former more commonly used to purify BEVs, whereas the latter is most frequently used for eukaryotic EVs (Akbar et al., 2022; Collins et al., 2021). These methods each have advantages and disadvantages (Hong et al., 2019; Konoshenko et al., 2018); a major strength of both methods being their ability to sub‐fractionate heterogenous populations of BEVs by density and size which is important for the characterisation of distinct BEV subpopulations (Dauros Singorenko et al., 2017; Hong et al., 2019). Conversely, both methods remove only small amounts of contaminating proteins from BEV preparations (Hong et al., 2019).

An issue specifically impacting the BEV field is the absence of a standardised method for BEV isolation and purification (Ñahui Palomino et al., 2021), making it difficult to compare results between different studies as the methods used may affect the morphology, yield and purity of the preparations. This, in turn, may affect downstream experimental analyses and therapeutic applications. Therefore, there is a need to develop improved BEV purification methods to either understand their functions or for clinical therapeutic applications. For eukaryotic EVs, it is clearly established that the cell culture media used contain contaminants (An et al., 2018; Botha et al., 2022; Karimi et al., 2018; Wachalska et al., 2016; Xu et al., 2019) that can have an impact on EV activities (Brennan et al., 2020; Holcar et al., 2021). In contrast, very few studies have investigated the correlation between BEV purity and downstream analyses, with all focusing on bacteria grown in defined growth media, such as RPMI (Dauros Singorenko et al., 2017; Hong et al., 2019). Many pathogenic bacteria have fastidious growth requirements and thus must be grown in complex, non‐defined broth media containing animal‐derived products and supplemented with serum (Albertson et al., 1998; Omsland et al., 2008).

Here, we describe an improved protocol for BEV purification from a complex bacteriological culture medium, used to grow the fastidious human pathogen, *Helicobacter pylori*. The aim of the study was to determine whether the depletion of animal‐derived products in the culture medium may result in purer BEV preparations. Therefore, we compared the properties of BEVs isolated using this new protocol, in which bacteria were grown in Brain Heart Infusion (BHI) broth depleted of animal‐derived products (D‐BHI), with BEVs isolated from bacteria grown in a standard culture medium. BHI medium consists of proteose peptone, dextrose and infusions from calf brain and beef heart, which are the sources of essential growth factors, carbon, nitrogen, sulphur, amino acids and vitamins crucial for bacterial survival. Dextrose is also included as a source of energy (Rijal, 2022). We showed that the standard BHI medium contains ‘EV‐like particles’ (EVLPs) that interfere with proteomic analyses. *H. pylori* BEV samples prepared from bacteria grown under standard conditions in BHI medium contained greater particle numbers and protein concentrations when compared with preparations from bacteria grown in D‐BHI medium which had been depleted of many potential medium‐derived contaminants by filtration through 50,000 molecular weight cut‐off (MWCO) membranes. Importantly, proteomic analyses on BEV samples prepared from bacteria grown in D‐BHI medium detected 3.4‐fold more *H. pylori* proteins and 10‐fold fewer bovine‐derived proteins when compared with those prepared from standard BHI medium. Furthermore, 57 *H. pylori* proteins were uniquely detected in samples prepared from bacteria grown in D‐BHI. In conclusion, we have described an improved method for BEV isolation from a complex bacteriological medium that increases the purity of the isolated BEVs and reduces the potential confounding effects of contaminants within the culture medium.

## 2 MATERIAL AND METHODS

### 2.1 Culturing of cell line and H. pylori

Human AGS gastric cancer cells were maintained as previously described (An et al., 2018). *H. pylori* strain B128 7.13 was routinely cultured on Horse Blood Agar (HBA) containing Blood Base Agar No. 2 (Oxoid, Thermo Fisher Scientific, MA, USA), 8% v/v horse blood (Australian Ethical Biologicals) and Skirrow's selective supplement as previously described (Ferrero et al., 1994). Bacteria were incubated at 37°C under microaerobic conditions (Campygen, Oxoid, Thermo Fisher, MA, USA).

For growth in liquid broth, *H. pylori* were inoculated to an optical density (OD_600_) of 0.05 in either standard Brain Heart Infusion broth (BHI; BD Bacto) or D‐BHI, achieved by 50,000 MWCO filtration (Sartorius, Gottingen, Germany) supplemented with 0.2% (w/v) β‐cyclodextrin (Sigma‐Aldrich, MO, USA) and Skirrow's selective supplement (Ferrero et al., 1994). Cultures were incubated for 17 hours (mid‐exponential phase) under microaerobic conditions (Campygen, Oxoid, Thermo Fisher, MA, USA) at 37°C while shaking at 120 rpm.

### 2.2 Bacterial growth

Mid‐exponential phase culture of *H. pylori* was grown in either standard BHI or D‐BHI as above. OD_600_ readings and viable counts (CFU/mL) were determined at 0 and 17 h. Viable counts were examined by inoculating dilutions of *H. pylori* cultures on HBA plates for 5 days under microaerobic conditions at 37°C.

### 2.3 BEV isolation and purification

**FIGURE 1:**
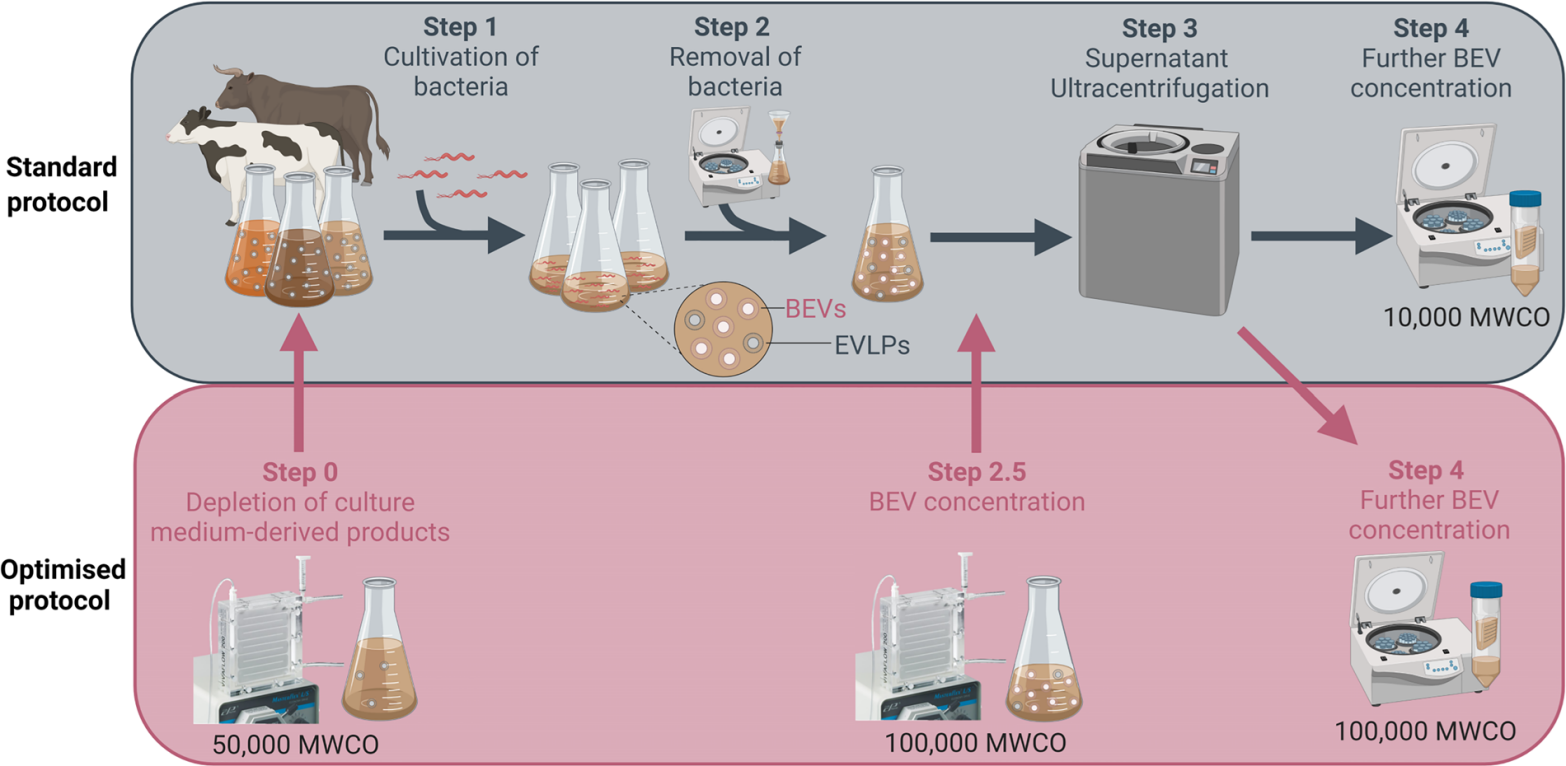
The standard and improved BEV purification methods used in the study. The standard BEV purification method consists of overnight incubation of bacteria in culture medium (step 1), followed by their removal by low‐speed centrifugation and sterile filtration (step 2), ultracentrifugation of cell‐free culture broth (step 3) and concentration of BEVs by low molecular weight filters (10,000 MWCO; step 4). The optimised BEV purification method includes pre‐depletion of culture medium‐derived products by ultrafiltration (50,000 MWCO; step 0). The resulting ‘flow‐through’ fraction is then used to culture the bacteria instead of the standard medium. An additional ultrafiltration (100,000 MWCO) was included to concentrate cell‐free culture broth before ultracentrifugation (step 2.5). Concentration of BEVs was then performed using 100,000 MWCO filters instead of those of low molecular weight, as in the standard protocol (step 4). The figure was created by BioRender.com.

For the standard method, *H. pylori* BEVs were isolated as described previously (Alves et al., 2015) (Figure 1). *H. pylori* was grown in standard BHI broth as described above. Bacterial cells were removed from the cultures by low‐speed centrifugation (4000 × *g* for 20 min at 4°C), followed by filtration through 0.22 μM pore membranes (Corning, Mulgrave, Victoria, Australia). Cell‐free cultures were subjected to ultracentrifugation (100,000 × *g* for 2 h at 4°C), and the supernatants discarded, leaving just 5 mL above the BEV pellets. These pellets were resuspended in the 5 mL of the remaining supernatants before concentration by 10,000 MWCO Amicon filters at 4000 × *g* at 4°C. The concentrated BEVs were then washed three times, each with 3 mL of PBS, using Amicon filters. BEV preparations were used immediately or stored at −20 C.

The optimised protocol of BEV isolation consisted of a few changes to the standard method (Figure 1). Bacteria were grown in BHI depleted of animal‐derived product (depleted‐BHI) which was achieved by tangential flow filtration (TFF) using a 50,000 MWCO Viva flow (Sartorius, Gottingen, Germany). Before ultracentrifugation, cell‐free cultures were concentrated by TFF using a 100,000 MWCO Viva flow (Sartorius, Gottingen, Germany) to reduce the culture volume 6‐fold. All BHI medium and cell‐free supernatants that had been concentrated by TFF were sterilised by filtration through 0.22 μM pore membranes before downstream use. Ultracentrifuged BEV preparations were collected, concentrated and washed as described above, but using a higher cut‐off pore size of 100,000 MWCO (Amicon). The higher filter pore size was to allow the exclusion of larger molecular weight products in the culture medium, so as to improve the purity of the BEV preparations and reduce the medium components that might interfere with downstream analyses.

### 2.4 Characterisation of EVs

#### 2.4.1 Electron microscopy

BEVs were visualised by negative staining electron microscopy where samples were absorbed onto carbon support grids for 3 min, then stained with 1% uranyl acetate for three intervals of 30 s. Grids were examined on the FEI Tecnai Spirit electron microscope (Fei company, Oregon, USA) with an accelerating voltage of 120 kV. Images were taken at 67,000 × magnification at random across the grids. Three fields were imaged for each biological replicate of BEVs.

#### 2.4.2 Nanoparticle tracking analysis (NTA)

The size distribution and the number of particles were determined by NTA using NanoSight NS300 (Malvern Panalytical, Malvern, UK). BEV preparations were diluted 1:5000 in pre‐degassed PBS whereas concentrated (10,000 MWCO filters) uninoculated broth media were diluted 1:10. Particles were detected at camera level 12 and recorded for five 30 s reads. The recordings were processed by NanoSight 3.4 software using a detection threshold of 4.

#### 2.4.3 Protein quantification

The concentration of proteins in the samples was quantified using the Qubit protein assay kit (Thermo Fisher Scientific, MA, USA) and a Qubit 1.27 Fluorometer, according to the manufacturer's guidelines. The purity of BEV preparations was estimated with a particle/protein ratio as previously described (Webber & Clayton, 2013).

$$Purity\left(Particles/protein\right)=\frac{Particles/mL}{\mu g/mL}$$

#### 2.4.4 Mass spectrometry analysis

Samples were processed using sodium deoxycholate (SDC) solubilisation as described previously (Huang et al., 2020). Using a Dionex UltiMate 3000 RSLC nano system equipped with a Dionex UltiMate 3000 RS autosampler, an Acclaim PepMap RSLC analytical column (75 μm × 50 cm, nanoViper, C18, 2 μm, 100Å; Thermo Scientific) and an Acclaim PepMap 100 trap column (100 μm × 2 cm, nanoViper, C18, 5 μm, 100Å; Thermo Scientific), the tryptic peptides were separated by increasing concentrations of 80% acetonitrile (ACN)/0.1% formic acid at a flow of 250 nL/min for 158 min and analysed with an Orbitrap Exploris 480 mass spectrometer equipped with a FAIMS module (Thermo Fisher Scientific, MA, USA).

The instrument was operated in data‐dependent acquisition mode to automatically switch between full scan MS and MS/MS acquisition. Each survey full scan (m/z 3750–1200; FAIMS voltage –45 V) was acquired in the Orbitrap with a resolution of 60,000 (m/z 200) after accumulating ions with a normalised AGC (automatic gain control) target of 300% and an automated maximum injection time. The 30 most intense multiply charged ions (*z* ≥ 2) were sequentially isolated and fragmented in the collision cell by higher‐energy collisional dissociation (HCD) with a resolution of 15,000, an AGC target of 50% and an automated maximum injection time. Dynamic exclusion was set to 20 s.

The raw data files were analysed with MaxQuant software v1.6.5.0 (Tyanova et al., 2016) and Andromeda search engine (Cox et al., 2011) to obtain protein identifications and their respective label‐free quantification (LFQ) values using standard parameters. A protein database was constructed by combining data for *H. pylori* strain B128 7.13 (accessed July 2022) and *Bos taurus* (Swissprot and TREMBL, accessed June 2021). The proteomics data were further analysed with LFQ‐Analyst (Shah et al., 2020). The relative abundance of *H. pylori* and bovine proteins was determined by calculating the amounts of ion intensity assigned for each BEV preparation. Statistical significance was determined using the package *Limma* with significance set at an adjusted *p*‐value = 0.05 (Benjamin‐Hochberg corrected) and log_2_‐fold change of 1.

### 2.5 Gel electrophoresis and immunoblot analysis

#### 2.5.1 Sample preparation

To examine the protein content of *H. pylori* grown in the different BHI broths, bacterial lysates were prepared from sonicating mid‐exponential phase *H. pylori* cultures. Bacterial lysis was achieved by sonication on ice for 6 × 30 s at 50% amplitude using a 4710 series Ultrasonic Homogeniser (Antylia Scientific company, IL, USA). To examine the uninoculated BHI broth for eukaryotic EV markers, standard and D‐BHI (prepared as above) were concentrated with 10,000 MWCO Amicon filters (Merck, MA, USA). All samples were resuspended in 4x Laemmli sample buffer (Bio‐Rad, CA, USA) and heated at 70°C for 10 min.

#### 2.5.2 SDS–PAGE electrophoresis

All samples were separated by NuPAGE 4%–12% Bis‐Tris 1.5 mm mini protein gels (Thermo Fisher Scientific, MA, USA). Bacterial whole cell lysates and BEV preparations were loaded at 7–15 μg or 30–40 μg per well for analysis by Western blotting or Coomassie blue staining, respectively. Uninoculated BHI broths and the positive controls used (supernatants and whole cell lysates) were loaded at 50 μg per well. After electrophoresis, the gels were either stained with Coomassie Brilliant blue R (Sigma‐Aldrich, MO, USA) or transferred onto 0.45 μm immobilon‐PVDF membranes (Merck, NJ, USA) at 100 V for 70 min.

#### 2.5.3 Immunoblotting

Membranes were stained with Ponceau S (Sigma‐Aldrich, MO, USA) before blocking with 5% skim milk for 1 h. Blots were incubated overnight at 4°C with primary antibodies (all diluted 1:1000) in 1% skim milk. Rabbit antibodies were generated ‘in‐house’ to either: whole *H. pylori* bacteria (Ferrero et al., 1994); *H. pylori* BEVs prepared from bacteria grown under the standard culture conditions and ultracentrifuged, as described here (Kaparakis et al., 2010); or recombinant UreA (Ferrero et al., 1994). Commercial antibodies were as follows: rabbit anti‐human EpCAM (ab71916, Abcam, Cambridge, UK), mouse anti‐Alix fusion protein (3A9, MA1‐83977, Thermo Fisher Scientific), rabbit anti‐human CD9 (PA5‐85955, Thermo Fisher Scientific) and mouse anti‐bovine CD9 (IVA50, MA1‐19301, Thermo Fisher Scientific). Blots were washed three times with TBS‐Tween (0.05% v/v) and incubated for 2 h at room temperature with either rabbit anti‐mouse‐IgG‐HRP (31450, Thermo Fisher Scientific) or goat anti‐rabbit‐IgG‐HRP (31460, Thermo Fisher Scientific) diluted 1:2000 in 1% skim milk. Membranes were developed with an Amersham ECL immunoblotting reagent (GE Healthcare, IL, USA) and imaged using an Amersham Imager 680 reagent (GE Healthcare, IL, USA).

### 2.6 Cell co‐culturing assay

Human AGS gastric cells were seeded in 12 well plates at 1 × 10^5^ cells/mL and serum‐starved as described previously (Tran et al., 2018). Cells were stimulated with increasing doses (25, 50 and 100 μg/mL) of BEVs for 24 h. As negative controls, cells were either left untreated or stimulated with 50 μg of concentrated (10,000 MWCO) uninoculated BHI, retentate or D‐BHI. Culture supernatants were collected at 24 h post‐stimulation and examined for IL‐8 production using a human IL‐8 ELISA kit (BD Biosciences, NJ, USA), as per the manufacturer's instructions. Absorbance values were measured at 450 nm with a FLUOStar Optima microplate reader (BMG Labtech, Ortenberg, Germany). Cytokine levels were determined by linear or 4‐parameter fit analysis.

### 2.7 Statistical analysis

GraphPad Prism v. 9.4.1 (GraphPad Software, CA, USA) was used for graphical representation and statistical analysis of the data. NTA and ELISA results were analysed by one‐way ANOVA followed by Tukey multiple comparison test. Particle/protein ratio results were compared by unpaired *t*‐test. Values of *p <* 0.05 were considered statistically significant.

## 3 RESULTS

### 3.1 Preparation of BEVs from H. pylori bacteria grown in either BHI or D‐BHI medium

To determine whether the depletion of certain culture media components might result in an improved method of BEV purification from complex media, *H. pylori* bacteria were grown in either standard BHI or D‐BHI media, as described in Figure 1. To produce D‐BHI medium, animal‐derived products were removed by filtration through 50,000 MWCO membranes (Step 0; Figure 1). The resulting filtrates (D‐BHI) and retentate fractions, as well as standard BHI medium, were then used to grow *H. pylori*. After overnight incubation, bacteria were removed from the spent culture broths, with the cell‐free supernatants subjected to ultracentrifugation and concentration using the methods described above (see Section 2). In the optimised protocol, two additional steps were included to concentrate the BEV preparations (Steps 2.5 and 4; Figure 1). The D‐BHI and retentate fractions supported bacterial growth to similar levels as standard BHI medium (Figure S1a,b). The bacteria grown in these media also had similar protein profiles, as determined by SDS–PAGE and Western blotting analysis (Figure S1c–e). Consistent with this finding, *H. pylori* BEVs isolated from bacteria grown in either BHI or D‐BHI also had similar protein profiles (Figure S1f).

**FIGURE 2:**
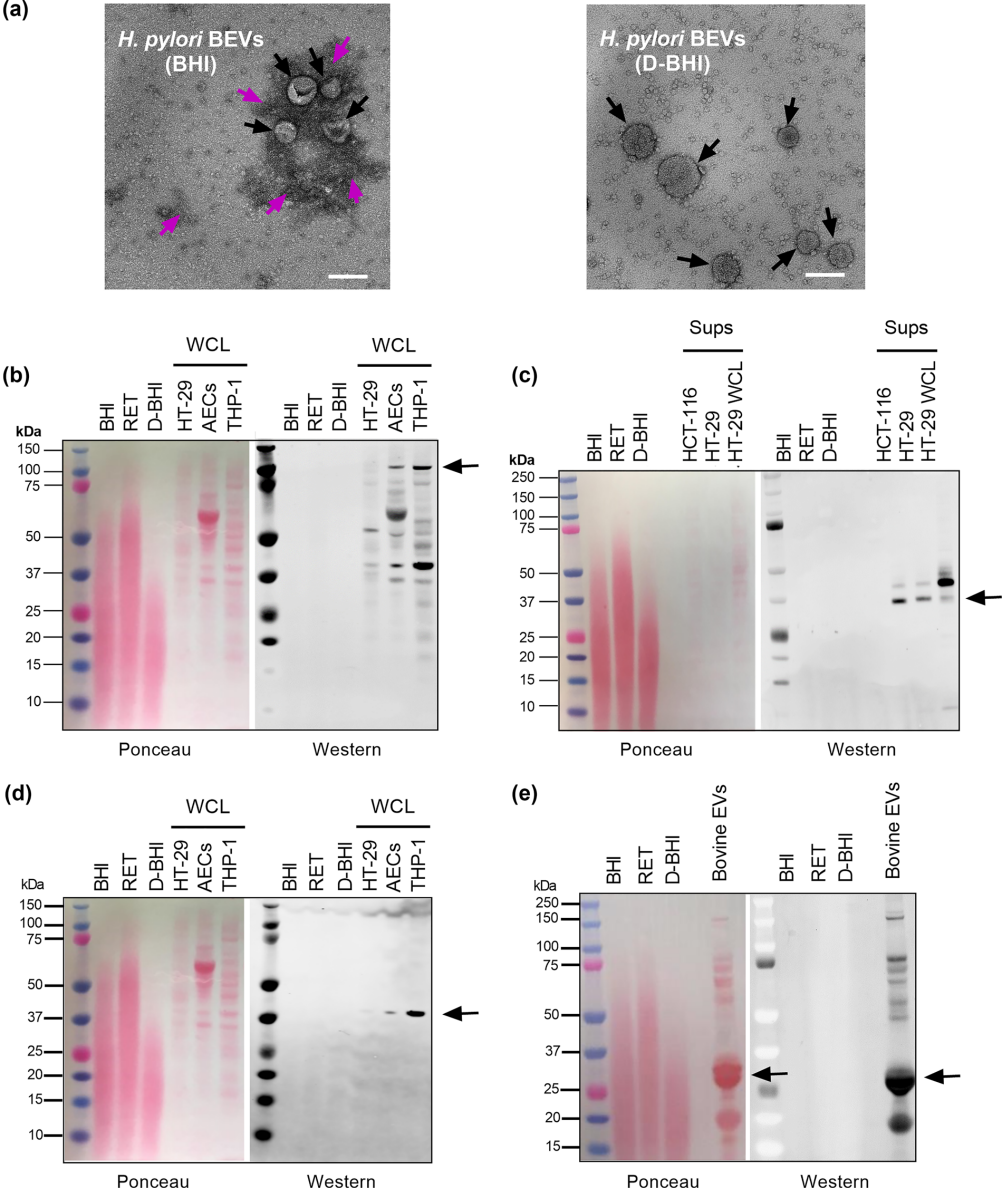
Morphology of BEV preparations. (a) Transmission electron microscopy (TEM) images of BEVs isolated from *H. pylori* grown in either standard BHI or depleted‐BHI (D‐BHI) media. Black arrows indicate BEVs, while purple arrows indicate culture‐derived extracellular vesicle‐like particles (EVLPs) or protein aggregates. TEM images are representative of at least three fields from *n* = 3 biological samples. Scale bar = 100 nm. (b–e) Uninoculated BHI, retentate (RET) fraction and D‐BHI collected from ultrafiltration (50,000 MWCO), followed by concentration (10,000 MWCO), were loaded onto SDS–PAGE gels (50 μg). As positive controls, we used supernatants (Sups) and whole cell lysates (WCL) from HT‐29, THP‐1, HCT‐116 cell lines, amniotic epithelial cells (AECs) and bovine EVs. Proteins were transferred onto membranes by Western blotting, stained with Ponceau S and then reacted against antibodies to various EV markers, as follows: (b) Alix, (c) Epcam and (d, e) CD9. Anti‐CD9 antibody used in (d) is also reactive to bovine proteins. *n* = 3 biological replicates.

Transmission electron microscopy of the BEVs that had been prepared from either BHI or D‐BHI media contained vesicles of similar morphology and sizes (Figure 2a). Dark, amorphous structures were, however, seen surrounding the BEVs prepared from standard but not D‐BHI medium. These structures were hypothesised to be EVLPs that co‐isolated with the BEVs prepared from BHI medium. To address this question, we analysed uninoculated BHI, retentate and D‐BHI preparations, which had been concentrated using 10,000 MWCO filters (Figure 1), and blotted these preparations against the eukaryotic EV markers Alix, Epcam and CD9 (Figure 2b–e). The three types of preparation were all negative for these EV markers. As positive controls, we included whole cell lysates and supernatants of a range of cell lines (Figure 2b–d). Milk‐derived bovine EVs were also tested to confirm that the anti‐CD9 antibody used was reactive against bovine proteins (Figure 2d). These findings suggest that the EVLPs within BHI are not true EVs but most likely large protein aggregates.

### 3.2 *H. pylori* BEVs prepared from D‐BHI medium are similar to those from BHI medium

**FIGURE 3:**
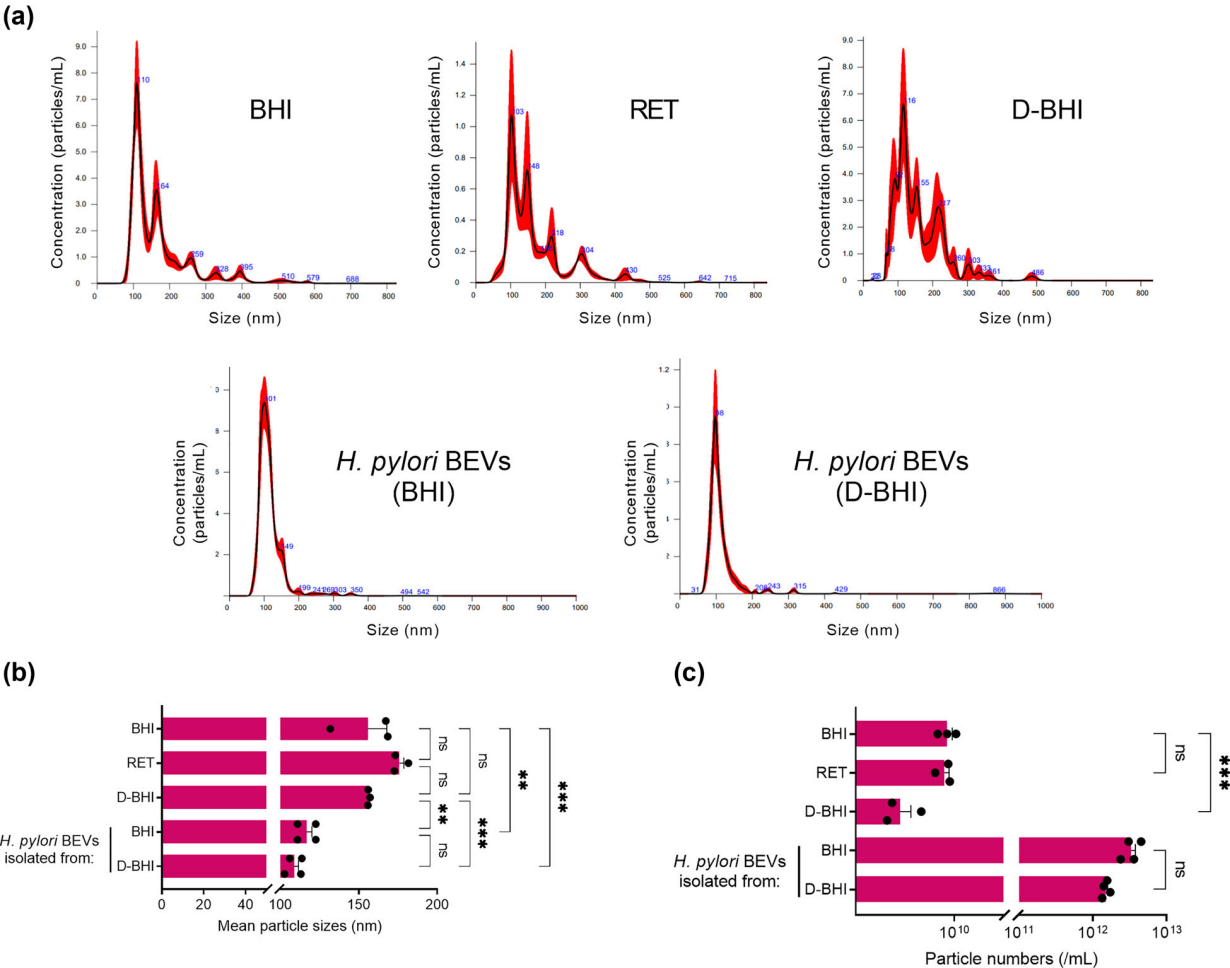
BEV preparations from bacteria grown in both standard and D‐BHI are similar in particle sizes and numbers. (a) Particle size distribution and numbers of the BHI broth (diluted 1:10) and BEV preparations (diluted 1:5000). (b) Sizes and (c) concentrations of particles in these preparations. Groups were compared by one‐way ANOVA followed by Tukey multiple comparison test. ns = not significant. **p < 0.01, ***p < 0.001 Error bars: Mean ± SEM. *n* = 4 biological replicates.

Next, we used nanoparticle tracking analyses (NTA) to determine the sizes and concentrations of EV particles from bacteria grown in either BHI or D‐BHI media (Figure 3). Additionally, we analysed uninoculated BHI medium, as well as the filtrates (D‐BHI) and retentate fractions, which had undergone the concentration steps detailed in Figure 1. The uninoculated media contained particles, which we designated EVLPs, whose size was much more broadly distributed than those of particles in the BEV preparations, for which particle size distributions seemed similar (Figure 3a). Some broadening of the peak was, however, observed for the BHI‐prepared BEVs when compared with those prepared from D‐BHI medium. Importantly, the mean particle size of BEVs prepared from D‐BHI medium (approximately 110 nm) was not significantly different to those prepared from standard BHI medium, but smaller than the EVLPs within the uninoculated media which had a mean size of 150 nm (*p* < 0.001) (Figure 3b). Particle numbers in uninoculated D‐BHI medium were significantly lower than in standard BHI medium (*p* < 0.001), but similar for BEV preparations in the two types of BHI media (*p* = 0.1584) (Figure 3c). Analysis of the NTA results revealed that the proportion of EVLPs in the D‐BHI‐derived BEV samples was 0.14%–0.32%, versus 0.029%–0.14% for those prepared from standard BHI medium. Therefore, although EVLPs are detected within BHI by NTA, these do not significantly contribute to total particle numbers in BEV preparations. Taken together, we have shown that D‐BHI that has been depleted of EVLPs and many of the animal‐derived components within the medium supports good *H. pylori* growth and, moreover, that the BEVs prepared from this medium share similar morphologies and sizes to those prepared from standard BHI medium.

### 3.3 BEV preparations from D‐BHI medium are enriched in *H. pylori* proteins and have lower levels of medium‐derived contaminants

**FIGURE 4:**
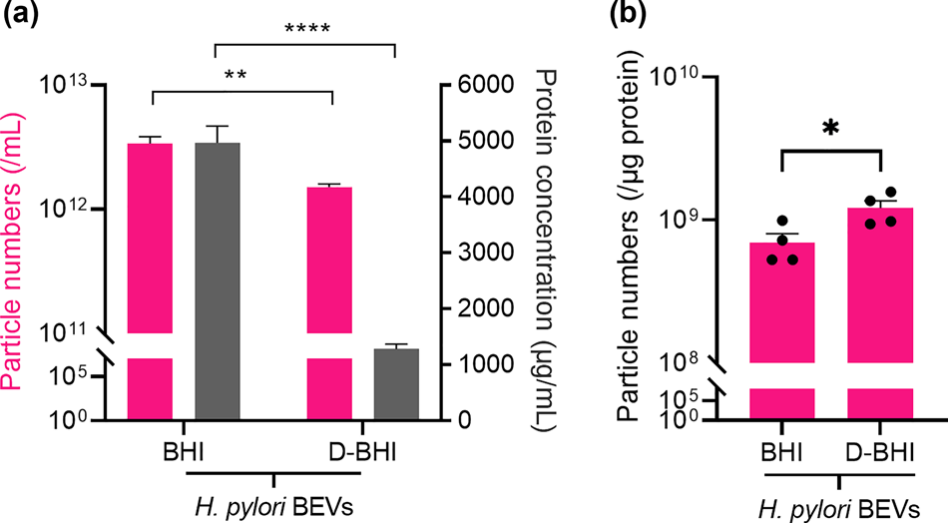
D‐BHI prepared BEVs have higher particle/protein ratios compared with those from standard BHI medium. (a) The particle numbers (pink) and protein concentrations (grey) of BEVs isolated from *H. pylori* bacteria grown in standard and D‐BHI media. (b) Particle/protein ratios as a measure of the relative purity of *H. pylori* BEVs prepared from the two media. Groups were compared by unpaired t‐test. ns = not significant. *p < 0.05, **p < 0.01, ****p < 0.0001. Error bars: Mean ± SEM. *n* = 4 biological replicates for both particle and protein concentration.

To investigate the purity of BEVs prepared from *H. pylori* bacteria grown in either BHI or D‐BHI, we determined the particle/protein ratios by NTA and Qubit assay, respectively (Webber & Clayton, 2013). BEVs prepared from bacteria grown in BHI medium contained approximately 74% more protein than D‐BHI‐prepared BEVs (mean = 4967 ± 299 vs. 1280 ± 94 μg/mL, respectively; *p* < 0.0001) (Figure 4a). Furthermore, *H. pylori* BEVs prepared using D‐BHI medium had significantly higher mean particle/protein ratios when compared with those obtained from standard BHI medium (1.21 × 10^9^ ± 1.5 × 10^8^ vs. 6.92 × 10^8^ ± 1.1 × 10^8^ particles/μg protein, respectively (*p* < 0.05) (Figure 4b). These results suggest that BEV preparation using D‐BHI medium resulted in samples with fewer protein contaminants.

**FIGURE 5:**
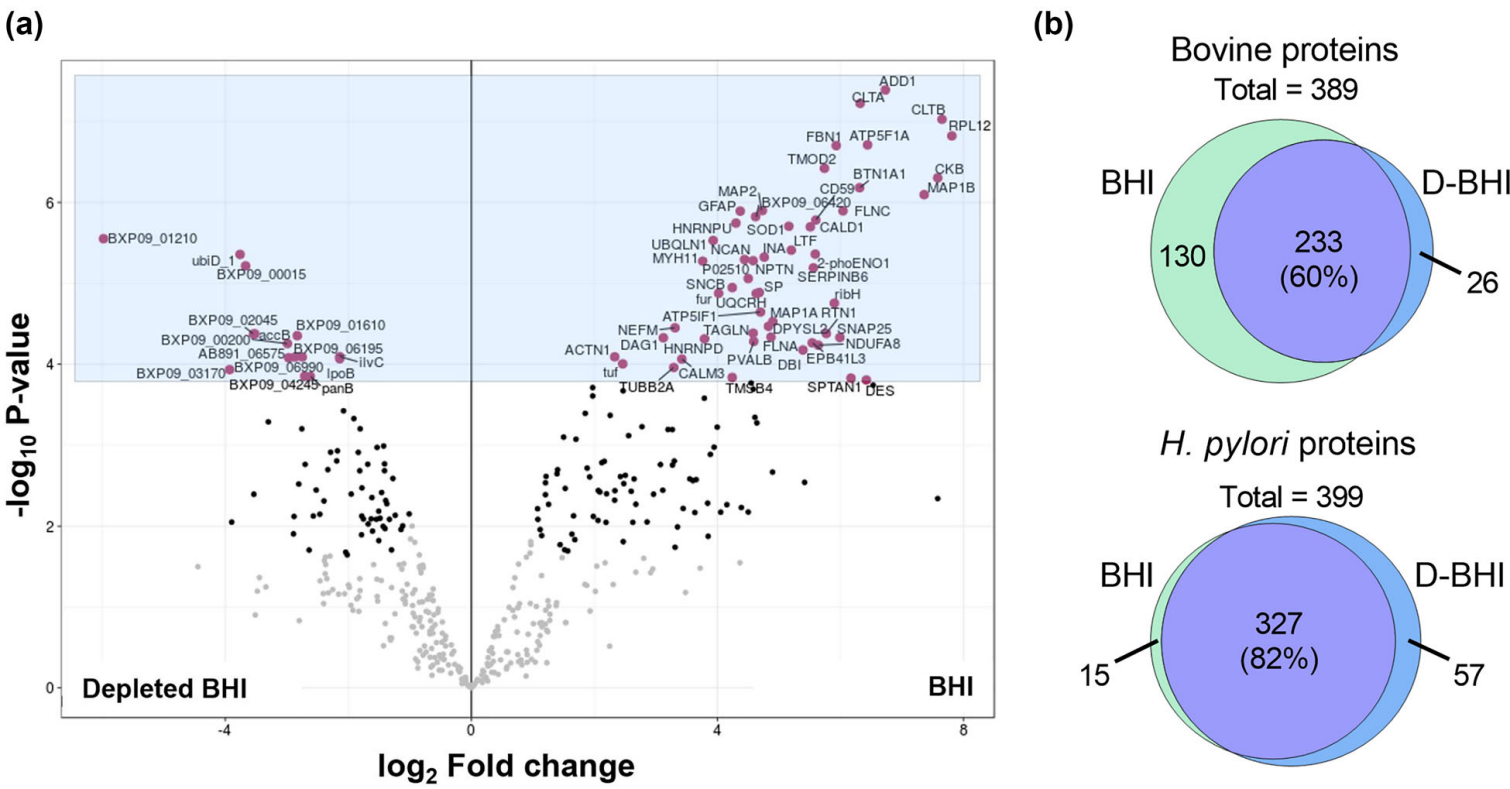
BEV preparations from depleted BHI are enriched in *H. pylori* proteins and contain fewer bovine proteins compared with those from BHI medium. Proteomic analyses were performed on BEVs isolated from *H. pylori* grown in either BHI or depleted BHI (D‐BHI) medium. (a). Volcano plot showing the enrichment of bovine (left) and *H. pylori* (right) proteins in BEVs prepared from bacteria grown in BHI or D‐BHI medium, respectively. The black dots represent proteins that had been significantly enriched in the sample. (b). A Venn diagram of bovine and *H. pylori* protein numbers in BEV samples prepared from the two media. *n* = 4 biological replicates.

Next, we performed proteomic analyses on BEV preparations using the two methods (Figure 1) to further determine the degree of sample purity and whether this may affect the detection of *H. pylori* proteins in these preparations. The volcano plot in Figure 5a (right panel) demonstrates an increased number of bovine proteins detected in BEVs prepared from bacteria that had been grown in BHI, whereas *H. pylori* proteins were enriched in BEV samples prepared from D‐BHI medium (Figure 5a; left panel). The top 15 most significantly abundant *H. pylori* proteins (based on the fold‐changes in protein amounts between BHI‐ and D‐BHI‐prepared BEVs) comprised predominantly membrane‐associated and metabolic proteins, as well as one virulence protein (OipA) (Table 1). This finding is consistent with previous proteomic studies on *H. pylori* BEVs (Turner et al., 2018; Zavan et al., 2019).

**TABLE 1:** The top 15 *H. pylori* proteins that were significantly enriched in BEV preparations isolated using depleted BHI media versus standard BHI

Protein name	Gene ID	Gene name	Fold increase (BHI vs. D‐BHI)	*p*‐value (BHI vs. D‐BHI)
Cytochrome c‐553	*BXP09_01210*	*Cytc553*	5.98	2.81E‐06
Acyl‐CoA thioesterase	*BXP09_03170*	*vdlD*	3.93	1.17E‐04
Outer inflammatory protein (OipA)	*oipA*	*oipA*	3.9	8.91E‐03
Flavin prenyltransferase (UbiX)	*ubiD_1*	*ubiX*	3.76	4.39E‐06
Competence protein	*BXP09_00015*	*HPB8*_2	3.67	6.09E‐06
Glutamate dehydrogenase	*BXP09_02045*	*gdhA*	3.54	4.24E‐05
Outer membrane beta‐barrel protein	*BXP09_00395*	*HPB8*_83	3.54	4.03E‐03
Biotin carboxyl carrier protein of acetyl‐CoA carboxylase	*accB*	*accB*	3.52	4.22E‐05
Endolytic peptidoglycan transglycosylase (RlpA)	*rlpA*	*rlpA*	3.3	5.13E‐04
TolC family protein	*BXP09_00200*	*HPB8*_41	2.99	5.57E‐05
Cystathionine gamma‐synthase	*BXP09_06990*	*metB*	2.97	8.34E‐05
Transporter	*BXP09_05025*	*ompP1*	2.89	1.25E‐02
Acetyl‐CoA C‐acetyltransferase	*BXP09_04250*	*atoB*	2.88	7.58E‐03
DUF904 domain‐containing protein	*AB891_06575*	*HPB8*_435	2.87	8.19E‐05
Outer membrane protein	*BXP09_01610*	*HPB8*_342	2.83	4.45E‐05

Further analyses of the BEVs identified a total of 389 bovine and 399 *H. pylori* proteins in these preparations (Figure 5b). (A complete list of the proteins is presented in Supplementary Data 1.) Of the total bovine proteins identified by mass spectrometry, 60% (corresponding to 233/389 proteins) were detected in both BHI‐ and D‐BHI‐prepared BEVs, with 130 uniquely detected within the former versus only 26 in the latter. In contrast, 82% (327/399) of the total *H. pylori* proteins identified were present in both BEV preparations, whereas 57 were uniquely found in BEVs prepared from D‐BHI when compared with 15 unique to BHI‐prepared samples (Figure 5b). Calculation of the *H. pylori*/bovine protein ratios shows that there were 5‐fold higher numbers of *H. pylori* proteins in BEV preparations from bacteria grown in D‐BHI medium than in those from BHI medium (Supplementary Data 2). Furthermore, examination of the unique assignable protein areas in the two groups shows that the BEVs prepared from D‐BHI medium had 10‐fold fewer bovine and 3.4‐fold more *H. pylori* proteins than those prepared from BHI medium (Supplementary Data 2). In summary, the new method of BEV purification described here allows enhanced detection of bacterial proteins in BEVs prepared from complex culture media containing a range of animal‐derived products.

### 3.4 H. pylori BEVs prepared from D‐BHI have similar effects on epithelial cells to those from BHI medium

**FIGURE 6:**
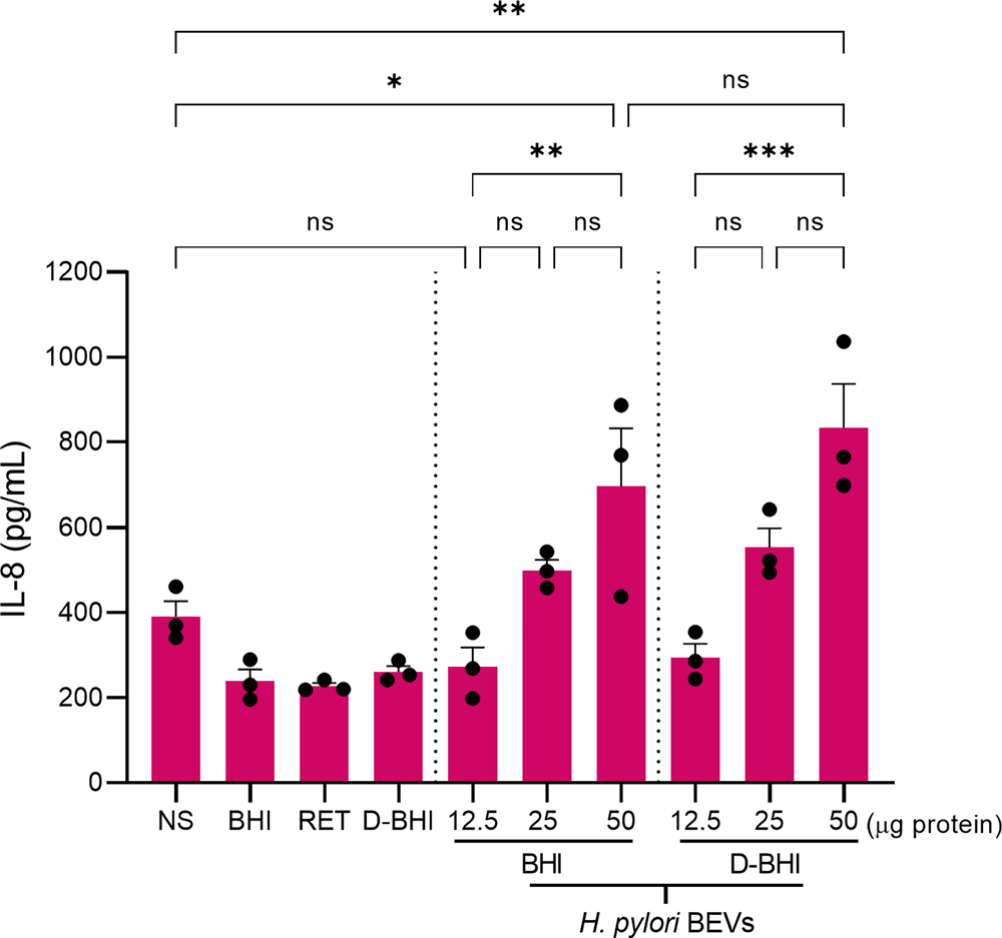
BEVs isolated from D‐BHI induced similar IL‐8 production in AGS cells as those from BHI medium. AGS cells were stimulated with increasing doses of BEVs isolated from *H. pylori* grown in either BHI or D‐BHI. As negative controls, non‐stimulated (NS) cells and cells stimulated with 50 μg uninoculated samples of BHI, retentate fraction or D‐BHI were included. At 24 h post‐stimulation, supernatants were examined for IL‐8 levels by ELISA. Groups were compared using a one‐way ANOVA followed by Tukey multiple comparison test. ns = not significant. *p < 0.05, **p < 0.01, ***p < 0.001. Error bars: Mean ± SEM. *n* = 3 biological replicates.

As *H. pylori* BEVs are reported to induce the production of the proinflammatory chemokine, interleukin‐8 (IL‐8), in epithelial cells (Kaparakis et al., 2010), we next examined the IL‐8‐inducing abilities of BEVs isolated from bacteria grown in either BHI or D‐BHI media. For this, human gastric epithelial AGS cells were stimulated with increasing doses of BEVs isolated from the two types of BHI media. As negative controls, cells were either not treated with the BEVs or incubated with uninoculated aliquots of BHI, retentate fraction or D‐BHI (50 μg protein/sample). BEV doses of 50 μg protein, but not 12.5 and 25 μg, induced significantly more IL‐8 production when compared with non‐stimulated cells (*p* < 0.05) (Figure 6). No significant differences were, however, observed in dose‐responses for cells stimulated with BEVs prepared from bacteria grown in either BHI or D‐BHI medium. In summary, although *H. pylori* BEVs isolated from D‐BHI medium were enriched in *H. pylori* proteins and had fewer contaminating proteins than BEVs isolated from the standard BHI medium, no significant differences were observed in their IL‐8‐inducing activities in AGS cells.

## 4 DISCUSSION

It is well‐known in the eukaryotic EV field that contaminants from biological samples and growth media can co‐isolate with EVs and subsequently affect their biological functions (Brennan et al., 2020; Holcar et al., 2021; Whittaker et al., 2020). Indeed, MISEV2018 and various studies have emphasised that changes to culture media composition, especially the depletion of important factors, such as serum and iron, can affect the cellular behaviour and composition of released EVs (Bost et al., 2022; Hong et al., 2019; Théry et al., 2018). In contrast, the effects of media composition on bacterial EV isolation and functions have not yet been investigated in detail. Although many bacteria can grow in defined media, pathogenic bacteria are often fastidious in nature and require complex media containing animal‐derived products to grow (Albertson et al., 1998; Omsland et al., 2008). Here, we report an improved method for BEV purification from a complex bacteriological culture medium in which animal‐derived contaminants had been depleted. Furthermore, we compared the properties of the BEVs prepared from this depleted medium (D‐BHI) with BEVs isolated from bacteria grown in standard medium.

We first examined *H. pylori* growth in standard and D‐BHI media. Viable bacterial counts showed that D‐BHI medium supported similar levels of bacterial growth as the standard medium (Figure S1b). It is likely that in the D‐BHI medium, small molecules (<50 kDa) such as amino acids essential for *H. pylori* growth (Nedenskov, 1994), were retained during the depletion process.

Using TEM, BEVs prepared from standard BHI were visualised to contain dark, amorphous structures that were absent in those prepared from D‐BHI (Figure 2a). We initially hypothesised these structures to be EVLPs, however, immunoblotting of BHI broth alone shows the absence of eukaryotic EV markers like Alix, Epcam and CD9 (Figure 2b–e). Numerous studies have described similar structures and suggested these to be lipoprotein/protein aggregates that can be co‐isolated from biological samples or growth media (Comfort et al., 2021; Holcar et al., 2020; Witwer et al., 2013). Linares et al. (2015) have described these protein aggregates to be the glue that sticks EVs together. Despite also utilising ultracentrifugation, the absence of protein aggregates in TEM images of BEVs prepared from D‐BHI suggested that the depletion of culture media is beneficial in obtaining pure BEV preparations. This may solve the existing problems associated with ultracentrifugation and protein aggregation. Overall, the improved method of BEV isolation can produce purer BEV preparations compared with the standard method.

Next, we used NTA to characterise the BEVs prepared from standard and D‐BHI media. Both methods were found to produce BEVs of similar sizes and concentrations (Figure 3). The sizes observed are consistent with a previous study that found 16‐hour grown *H. pylori* produces BEVs ranging from 50 to 500 nm, with the majority of BEVs being 100–200 nm in size (Zavan et al., 2019). As protein aggregates and low‐density lipoproteins within eukaryotic EV preparations were suggested to be detected by NTA (Gardiner et al., 2013; Lehrich et al., 2021), we also concentrated and analysed uninoculated BHI medium, retentate fraction and D‐BHI broth. Particles within all three types of BHI media were found to be similar in size (150–175 nm) (Figure 3b). This study is the first to provide direct evidence for NTA detection of particles within bacteriological culture media. Despite the mean size of particles within uninoculated BHI and D‐BHI media being considerably larger than those in BEV preparations, these did not appear to contribute to the particle size distributions for BEV preparations. Although unexpected, this observation can be explained by their low numbers within BEV samples. Also, the concentration method used on uninoculated BHI broth, involving 10,000 MWCO filters, may have caused the proteins to aggregate (Arakawa et al., 2017). Together, macromolecular protein aggregates within complex bacteriological culture media can be detected by NTA, however, these were found to not significantly contribute to particle counts of BEV preparations.

The purity of BEVs isolated by the two methods was determined by calculating the particle/protein ratio, as suggested by Webber and Clayton (2013). BEVs prepared from *H. pylori* grown in D‐BHI were found to have a higher mean particle/protein ratio compared with those from the standard BHI (1.21 × 10^9^ ± 1.5 × 10^8^ vs. 6.92 × 10^8^ ± 1.1 × 10^8^ particle/μg, respectively *p* < 0.05) (Figure 4b). Although these data suggested that the use of D‐BHI medium resulted in EV preparations of higher purity, such a particle/protein ratio was considered by Webber and Clayton as indicative of a low‐purity preparation (Webber & Clayton, 2013). Those researchers, however, analysed EVs produced by cancer cell lines which can produce up to 2 × 10^13^ particles/mL compared with 3 × 10^12^ particles/mL produced by *H. pylori* (Webber & Clayton, 2013). It is likely that differences in the EV source and particle numbers released may influence particle/protein ratios. Another study comparing different separation methods for helminth EVs (which produce up to 8 × 10^11^ particles/mL) had mean particle/protein ratios similar to those reported here for *H. pylori* EVs (Borup et al., 2022).

To further determine the purity of EV preparations using the improved method, we performed proteomic analyses on BEV preparations from the two types of BHI media. Importantly, BEVs prepared from bacteria grown in the D‐BHI medium were enriched in proteins of bacterial origin, whereas BEVs prepared from standard medium contained many more proteins of bovine origin (Figure 5). Of the 57 *H. pylori* proteins that were detected in the BEVs prepared from D‐BHI medium, five were uncharacterised (Figure 5b and Supplementary Data 2). Based on the calculated *H. pylori*/bovine protein ratios, we found *H. pylori* proteins to be enriched 5‐fold with respect to those of bovine origin in *H. pylori* BEVs prepared from D‐BHI compared with those from standard BHI broth. Further examination of the unique assignable protein areas in BEVs prepared from the two groups, revealed 3.4‐fold more *H. pylori* proteins and 10‐fold fewer bovine proteins in BEVs prepared from D‐BHI medium compared with those from standard BHI (Supplementary Data 2). These proteomic data strongly suggest that the improved method, which allows the depletion of BHI‐derived proteins, resulted in an enhanced detection of BEV proteins by proteomics. As mass spectrometry is routinely used by many workers to qualitatively and quantitatively analyse BEV preparations, our findings highlight the importance of reducing media contaminants within these preparations that may interfere with the detection of BEV‐associated proteins. To our knowledge, this is the first study to report the impact of media‐derived contaminants on proteomic analyses of BEVs.

*H. pylori* EVs were shown to be strongly pro‐inflammatory in epithelial cell models (Kaparakis et al., 2010). As co‐isolated contaminants from culture media can alter the biological activities of eukaryotic EVs (Whittaker et al., 2020), we examined the ability of BEVs from bacteria grown in BHI or D‐BHI to induce IL‐8 production in the AGS epithelial cell line. We did not observe any significant differences in IL‐8 responses (Figure 6), suggesting that the BEV purification method had no effect on this property of the BEVs. Nevertheless, further studies are warranted to determine whether the improved BEV isolation method reported here may have an impact on other *H. pylori* BEV actions on host cells, such as micronuclei formation (Chitcholtan et al., 2008), cell proliferation (Ismail et al., 2003) or cell viability (Chew et al., 2021). It is also possible that different cell lines may react differently to medium‐derived contaminants within BEV preparations (Choi et al., 2021). These data highlight the importance of understanding the potential impact of medium‐derived contaminants in BEV preparations.

In conclusion, we have described an improved method of BEV purification that can successfully deplete animal‐derived components from complex culture media used to prepare BEVs. The BEVs prepared using this method were purer but otherwise shared similar morphologies, sizes, concentrations and IL‐8‐inducing abilities as BEVs prepared from the standard culture medium. Importantly, we also report for the first time that the animal‐derived components within complex bacteriological culture media co‐isolate with BEVs and propose that these components interfere with proteomic analyses. As BEVs are increasingly considered as promising candidates for vaccine development, targeted drug delivery and biomarkers (Michel & Gaborski, 2022; Wang et al., 2019), it is critical to understand the potential contaminants within BEV preparations so as to prevent potential deleterious effects on the host. Further refinement of the method described here, such as by the inclusion of either DGC or SEC procedures in the workflow, would be expected to improve the quality and reproducibility of BEV preparations.

## CONFLICT OF INTEREST STATEMENT

There were no conflicts of interest.

## Supporting information

supplementary information

supplementary information

supplementary information

## Data Availability

Data will be made freely available if requested.
