# Supplementary figures and images for: A new isolation method for bacterial extracellular vesicles providing greater purity and improved proteomic detection of vesicle proteins
Source: J Extracell Biol. 2023 Apr 25;2(5):e84. doi: 10.1002/jex2.84 (PMC11080860; doi:10.1002/jex2.84)

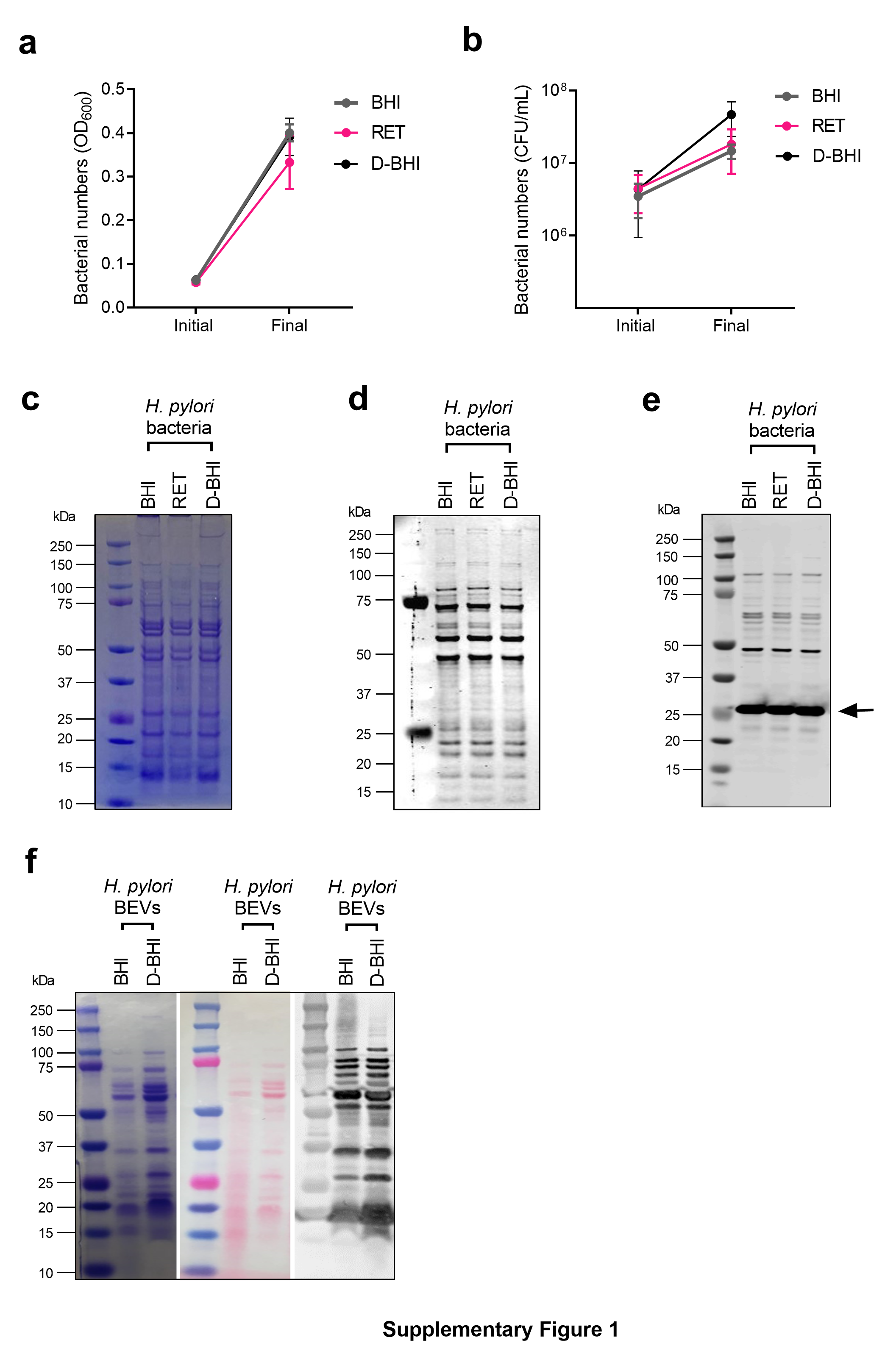

Supplement: Supplementary file 1 — supplementary information [file JEX2-2-e84-s001.tif]
